# Global Patterns and Prevalence of Dual and Poly-Tobacco Use: A Systematic Review

**DOI:** 10.1093/ntr/ntab084

**Published:** 2021-04-30

**Authors:** Daniel Tzu-Hsuan Chen, Charis Girvalaki, Enkeleint A Mechili, Christopher Millett, Filippos T Filippidis

**Affiliations:** Public Health Policy Evaluation Unit, School of Public Health, Imperial College London, London, UK; Clinic of Social and Family Medicine, School of Medicine, University of Crete, Heraklion, Greece; Clinic of Social and Family Medicine, School of Medicine, University of Crete, Heraklion, Greece; Department of Healthcare, Faculty of Public Health, University of Vlora, Vlorë, Albania; Public Health Policy Evaluation Unit, School of Public Health, Imperial College London, London, UK; Public Health Policy Evaluation Unit, School of Public Health, Imperial College London, London, UK

## Abstract

**Introduction:**

Improving understanding of the epidemiology of dual and poly-tobacco product use is essential for tobacco control policy and practice. The present study aimed to systematically review existing epidemiologic evidence on current dual and poly-tobacco use among adults globally.

**Methods:**

We systematically searched online databases for studies published up to June 30, 2020. We included quantitative studies with measures of nationally representative prevalence of current dual or poly-tobacco use among adults. Prevalence estimates for each country were extracted manually and stratified by WHO regions and World Bank income classifications.

**Results:**

Twenty studies with nationally representative prevalence data on current dual or poly-tobacco use in the adult population across 48 countries were included. Definitions of dual and poly-tobacco use varied widely. Prevalence of dual and poly-tobacco use was higher in low- and lower-middle-income countries compared to other higher-income countries. Current dual use of smoked and smokeless tobacco products among males ranged from 0.2% in Ukraine (2010) and Mexico (2009) to 17.9% in Nepal (2011). Poly-tobacco use among males ranged from 0.8% in Mexico (2009) and 0.9% in Argentina (2010) to 11.4% in the United Kingdom and 11.9% in Denmark in 2012. Dual tobacco use was generally higher in South-East Asia; poly-tobacco use was prevalent in Europe as well as in South-East Asia.

**Conclusions:**

This is the first systematic review of the prevalence estimates of dual and poly-tobacco use among adults globally. The results of the current study could significantly help health policy makers to implement effective tobacco control policies.

**Implications:**

This study demonstrates that dual/poly-tobacco use is common in many countries of the world, and highlights the need for in-depth exploration of this field in future studies, especially in high prevalence regions such as South-East Asian and European countries. In light of this, the global tobacco control community and health authorities should also agree upon a consistent operational definition of dual and poly-tobacco use to propel research and improve surveillance of dual/poly-use in health surveys for better communication and understanding of these phenomena.

## Introduction

Although the prevalence of adult cigarette smoking has declined globally since 1980,^1^ the increased diversity and growing market of alternative tobacco products have led to the growing prevalence of concurrent use of multiple tobacco products (ie, dual and poly-tobacco use) in recent years.^2^ Prevalence of multi-tobacco product use is high in the United States,^3^ especially among younger adults.^4^ In South-East Asia, where smokeless tobacco (SLT) use is popular, 7.5% of men were reported to be dual users.^5^

Users of multiple tobacco products face increased risks of tobacco-related diseases,^6^ greater nicotine dependence,^7^ and report weaker intention to quit.^8^ However, most regulations and tobacco control policies around the world tend to focus on manufactured cigarettes and may not be equally effective with non-cigarette tobacco products.^9^ Hence, improving understanding of the epidemiology of multiple-tobacco use may lead to more effective tobacco control policies. There is currently limited research on the global patterns of poly-tobacco use among smokers in different contexts, with existing studies largely focused on high-income countries and subgroups of interest.^10–12^ Few studies have assessed dual/poly-use among nationally representative samples and they have used variable definitions of dual and poly-tobacco use,^2,13^ which limits cross-country comparisons.

The present analysis aimed to systematically review existing epidemiologic evidence on current dual and poly-tobacco use among adults globally in order (a) to assess the best available prevalence estimates and (b) to review definitions of dual and poly-tobacco product use in the literature.

## Methods

We conducted a systematic review of studies published up to June 30, 2020. The PRISMA protocol^14^ was employed to guide the design of our review.

### Search Strategy

We searched Ovid for Medline, Embase, and Global Health for full text with language restrictions to English. We used “multiple” along with its synonyms and other variations describing the use of multiple tobacco products, such as “alternative,” “poly,” “concurrent,” and “dual” (detailed search strategy in Supplementary Table 1). In addition, we hand-searched the reference lists of included studies and used the Google Scholar function to identify additional articles.

### Eligibility Criteria

Studies were eligible for inclusion if they met the Population, Outcome, and Study design criteria of the PICOS framework^15^ as follows:

1. Population: nationally representative study sample of the adult population (>18 years old).
2. Outcomes: any prevalence measure of “current” dual (concurrent use of two tobacco products) or poly-tobacco use (concurrent use of more than two tobacco products). Tobacco products refer to products containing tobacco, including cigarettes (manufactured cigarettes or/and roll your own tobacco), pipe tobacco, cigars, cigarillos, SLT, and herbal tobacco products for smoking.
3. Study design: quantitative studies with prevalence estimates of tobacco use.

### Study Selection

Results from literature searches were merged and duplicates removed. Three authors (THC, CG, and EAM) independently screened the titles to identify potentially eligible publications, which were retrieved and their abstracts screened. The full text of publications deemed relevant were downloaded and examined rigorously. Disagreements regarding study eligibility were resolved by discussion between the three authors or with the assistance of a fourth author (FTF).

### Data Extraction

Three authors (DT-HC, CG, and EAM) worked in pairs to extract information from eligible studies, including study characteristics and prevalence data of: (1) each tobacco product assessed; (2) different combinations of tobacco products used; (3) dual and poly- tobacco product use; as well as the operationalized definition of dual and poly-tobacco use.

We extracted the prevalence of current tobacco product use defined as use in the past 30 days. When there were multiple publications with prevalence data from the same sample or study within a country, we extracted data from each publication, but used only the most recent estimate. We requested additional unpublished data from the corresponding authors where necessary.

### Data Analysis

Countries were categorized by WHO region (African, American, South-East Asian, European, Eastern Mediterranean, Western Pacific) and by country income level according to World Bank^16^ (high-income [HIC], upper-middle-income [UMIC], lower-middle-income [LMIC], and low-income [LIC]).

We present weighted, nationally representative prevalence estimates of dual and poly/multiple tobacco product use among the adult population of every country in eligible publications. The sample sizes were pooled for each country.

## Results

### Search Results and Study Characteristics

**Figure 1.**
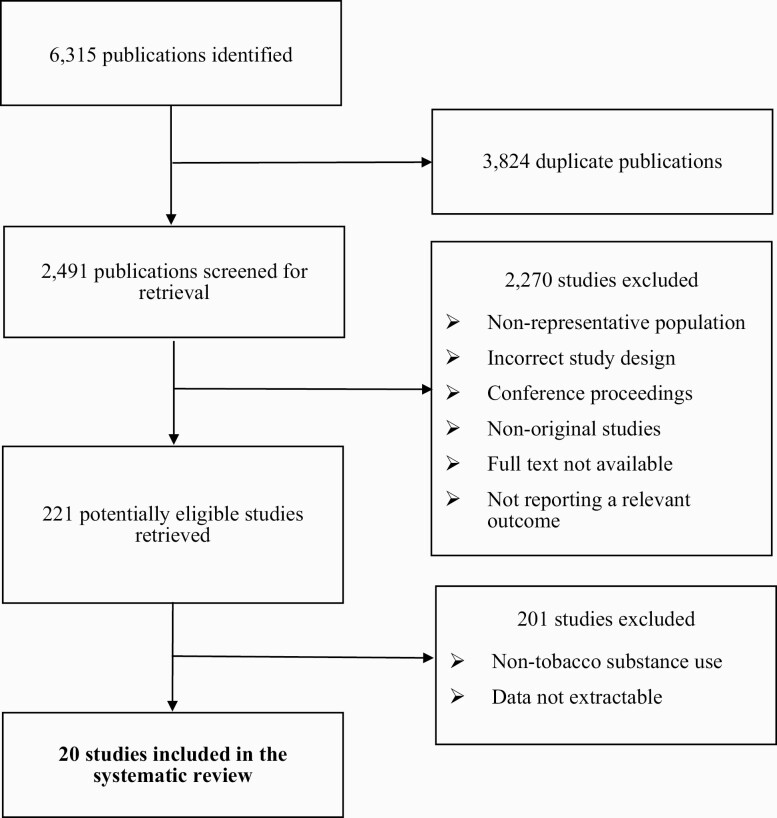
Flow diagram of the study selection.

In total, we identified 6315 publications. Following the exclusion of duplicate publications (*n* = 3824), 2491 potentially eligible studies were screened (Figure 1). The review included a total of 20^2,3,5,13,17–20,21–32^ studies with nationally representative prevalence data on current dual or poly-tobacco product use among adults across 48 countries, covering all six WHO regions with approximately 53% of the global population.^33^Supplementary Table 2 summarizes the characteristics of the included studies.

We obtained prevalence estimates from 1992 to 2018 in the United States and from 2005 to 2012 in other countries, with a total sample size of 2 165 464. Among the 20 studies, 14^2,3,13,22–32^ are nationally representative surveys of the United States, with almost one-third of the studies examining the National Health Interview Survey (NHIS); while the six non-US studies^5,17–20,21^ mostly used the Global Adult Tobacco Survey (GATS). All of the studies provided estimates on either dual use or poly (multiple) tobacco product use, and three studies^2,13,28^ reported both.

### Definition of Current Dual/Poly-Tobacco Product Use

We used a broader definition for current (monthly) tobacco use adapted from the Global Tobacco Surveillance System^34^ to capture both daily and non-daily users, including any use in the past 30 days across surveys. Current dual tobacco use among adults, also called “concurrent tobacco use”,^22,25–27^ was commonly determined in one of five ways (Supplementary Table 3): (1) Current use of one smoked and one SLT product^19–20,21^; (2) Current use of cigarettes and of one other tobacco product^2,22,32^; (3) Current use of cigarettes and SLT^25–27^; (4) Current use of any two tobacco products^13,28^; or (5) Current use of at least one smoked and at least one SLT product.^5^ A few studies defined current dual use as a specific combination of two tobacco products.^18,24,30,31^ Poly-tobacco use was commonly defined as consuming two or more tobacco products,^3,13,17,23,29^ in which case it includes dual use or in some studies, three or more products,^2,28^ also frequently referred to as “multiple tobacco product use”.^3,23,28,29^

### Global Prevalence of Dual and Poly-Tobacco Use

All 20 studies reported current single tobacco use among adults^2,3,5,13,17–20,21–32^ (Supplementary Table 4). As shown in Table 1, the prevalence of current dual use of smoked and SLT products ranged from 0.2% in Ukraine and Mexico^19^ among males to 17.9% in Nepal.^5^ In four South-East Asian countries (Indonesia, Maldives, Nepal, and Timor-Leste), where prevalence by sex was available, the proportion of dual use among males was substantially higher than among females by at least twofold.^5^ The combination of cigarette and hookah/waterpipe dual use was reported in five countries^3,18^: 0.3% in India, 0.4% in Egypt, 2.6% in Russia and Vietnam in 2009/2010, and 6.0% in the United States in 2013/2014. Five studies with data from 45 countries reported current poly-tobacco use among adults,^5,17–20^ showing widely varying prevalence across the globe, ranging from 0.8% in Mexico, 2009 to 11.9% in Denmark,^17^ 2012. Eleven studies in the United States reported prevalence of dual use from 1992 to 2016,^2,13,22,24–28,30–32^ and six studies estimated prevalence on poly-use from 1998 to 2018^2,3,13,23,28,29^ (Supplementary Table 5).

**Table 1.** Prevalence estimates of current dual/ poly-tobacco use among adults by country and region

			Dual/ poly-tobacco use (%)	
Region, country	Income level	Survey year	Dual use^a^	Poly-use^b^
South-East Asia				
Bangladesh^17,19^	LIC	2009	6.8	8.8
Indonesia^5,17^	LMIC	2012	M:0.2; F:0.1	4.6
India^d^^17–20^	LMIC	2009/2010	5.3	6.5
Maldives^5^	UMIC	2009	M:1.5; F:0.03	―
Nepal^5^	LMIC	2011	M:17.9; F:1.5	―
Timor-Leste^5^	LMIC	2009/2010	M: 2.6; F:0.8	―
Thailand^17^	UMIC		0.4	5.9
Western Pacific				
China^17,19^	UM	2010	0.4	2.1
Malaysia^17^	UM	2011	―	5.4
Philippines^17,19^	LMIC	2009	0.7	1.8
Viet Nam^d^^17–19^	LMIC	2010	0.1	3.5
Eastern Mediterranean				
Egypt^d^^17–19^	LMIC	2009	1.9	2.7
African				
Nigeria^17^	LMIC	2012	―	1.5
European				
Austria^17^	HIC	2012	―	10.6
Belgium^17^	HIC	2012	―	9.0
Bulgaria^17^	UMIC	2011	―	5.1
Cyprus^17^	HIC	2012	―	10.8
Czech Republic^17^	HIC	2012	―	9.1
Denmark^17^	HIC	2012	―	11.9
Estonia^17^	HIC	2012	―	7.9
France^17^	HIC	2012	―	10.4
Finland^17^	HIC	2012	―	9.8
Germany^17^	HIC	2012	―	9.5
Greece^17^	HIC	2012	―	10.5
Hungary^17^	UMIC	2012	―	7.8
Italy^17^	HIC	2012	―	5.3
Ireland^17^	HIC	2012	―	7.1
Luxembourg^17^	HIC	2012	―	8.7
Latvia^17^	HIC	2012	―	10.0
Lithuania^17^	HIC	2012	―	5.9
Malta^17^	HIC	2012	―	9.1
Netherlands^17^	HIC	2012	―	9.3
Poland^17,19^	HIC	2009	0.3	2.4
Portugal^17^	HIC	2012	―	6.2
Romania^17^	UMIC	2011	―	1.7
Russian Federation^d^^17–19^	HIC	2009	0.4	6.0
Spain^17^	HIC	2012	―	9.0
Sweden^17^	HIC	2012	―	5.2
Slovakia^17^	HIC	2012	―	4.7
Slovenia^17^	HIC	2012	―	5.2
Turkey^17,19^	UMIC	2008	―	3.7
Ukraine^17,19^	LMIC	2010	0.2	3.4
United Kingdom^17^	HIC	2012	―	11.4
American				
Argentina^17^	UMIC	2010	―	0.9
Brazil^17^	UMIC	2008	―	3.1
Mexico^17,19^	UMIC	2009	0.2	0.8
Uruguay^17,19^	HIC	2009	―	5.0
United States^d^^3,23^	HIC	2018	4.0^c^	3.7

M = male; F = female; LIC = Low-Income Countries; LMIC = Lower-Middle Income Countries; UMIC = Upper-Middle Income Countries; HIC = high-income countries; ― = data not available. Current use was determined as participants who had smoked or used the product in the previous 30 days. Table summarizes prevalence estimates weighted from various studies and surveyed years, and therefore, figures may not be directly comparable between each usage groups. Table presents most recent prevalence estimates available of each country and product use.

^a^Dual use: smoking + smokeless tobacco.

^b^Poly-use: consuming two or more tobacco products.

^c^Dual use of cigarettes+smokeless tobacco.

^d^Percentages of cigarette + hookah/waterpipe dual use: India = 0.3%; Vietnam = 2.6%; Egypt = 0.4%; Russian Federation = 2.6%; United States = 6.0%.

Among studies with consistent definition of dual/ poly-tobacco use, dual use of smoking and SLT was generally higher in countries in South-East Asia, America, and in Egypt; whereas poly-tobacco use defined as consuming two or more tobacco products was found to be highest in South-East Asia and the European countries, and relatively lower in Nigeria (Table 1). Dual and poly-use were also more prevalent in LICs and LMICs, compared to UMICs and HICs (Table 1).

Studies from the United States ^2,3,13,22–32^ found high prevalence of use of at least two (up to 37.8%) and at least three (up to 15.3%) products in 2013–2014^3^ (Supplementary Table 5).

## Discussion

### Main Findings

Results from our systematic review indicate that there were wide variations in dual and poly-tobacco use prevalence by region and income level. Regional differences in the prevalence of dual and poly-tobacco use may be influenced by cultural, social and economic factors.^35,36^ As cigarettes are almost ubiquitously popular around the globe, the proliferation of alternative tobacco products may open avenues for switching to cheaper products^37^ or smoking cigarettes in combination with other products,^38^ which may lead to high levels of poly-tobacco use transition to dual or poly-use. For example, SLT use is extremely prevalent and embedded in the culture in South and South-East Asia countries which accounts for almost 90% of the world users.^39,40^ In India, the prevalence of SLT use is higher than the prevalence of cigarette smoking.^39^ It is therefore not surprising that high prevalence of dual use was observed in these countries.^41^ This may also be relevant to Europe where use of alternative tobacco products and of dual/poly-use have been increasing.^37^ Considering the health consequences of such use patterns,^6^ dual and poly-tobacco use should be given higher priority in terms of regulation and research, especially in certain regions in the world with higher levels of use.

Our review revealed that published studies have been based on only 11 national surveys, with almost half on them in the United States. This is a small number compared to the abundant research on tobacco use worldwide. In contrast, during the screening process, we discovered a large number of studies on adolescent populations, including dual use with e-cigarettes. The small pool of data sources may reflect the fact that questions on dual/poly-tobacco use are not generally included in routine tobacco use surveys, and few studies have analyzed available datasets with dual or poly-tobacco use as an outcome of interest.

Definitions of dual and poly-tobacco use varied widely among the limited resources identified. How surveys define current use of tobacco products influences the reported prevalence of dual use, and can impact the prevalence estimates by 50-fold.^42^ Varied definitions also induce discrepancy across studies, which impacts comparability. Reaching a consensus regarding a consistent operational definition of “dual” and “poly-tobacco use” can facilitate global comparisons and strengthen tobacco use regulation.

### Strengths and Limitations

To our knowledge, this is the first systematic review of prevalence estimates of dual and poly-tobacco use across multiple countries worldwide. Our review covered adult population of countries in all six WHO regions and all income levels which allowed us to gain a comprehensive view of the regional differences in prevalence and patterns of use, although these may not be representative of the respective WHO regions or income levels when data come from a small number of countries. This review presented only the most recent data in each country, thus estimates may not be directly comparable between countries or reveal trends over time. Our review excluded dual and poly-use of drugs, other substances, and e-cigarettes, which further increase the complexity of tobacco use although it’s clear that such products and substances are and should be considered in tobacco control strategies. Finally, estimates rely on self-reported use.

### Policy Implications and Future Research

Improving surveillance of dual/poly-use in health surveys and related analyses is essential to monitor trends and use patterns. Data on product types, quantity, duration, and frequency of use of alternative tobacco products and dual/poly-use should be documented in all standard routine tobacco surveys to facilitate analysis and inform regulation. The global tobacco control community and health authorities should also agree upon a consistent operational definition and terminology of dual and poly-tobacco use. Longitudinal studies are also needed to allow investigation of transitions between single-product use and dual/poly-tobacco use and how these are affected by tobacco control policies. Existing tobacco control policies may not sufficiently address the complexities of poly-tobacco use^9,43^; therefore, new evidence can be used to tailor these policies to tackle the tobacco epidemic.

## Conclusion

Our systematic review provides prevalence estimates of dual and poly-tobacco use in 48 countries. Such information is essential for tobacco control policy and practice aiming to provide effective and comprehensive regulation that encompasses alternative tobacco products as well as dual and poly-use. As dual/poly-tobacco use is common in many regions of the world, having standardized questionnaires and shared definitions of dual/poly-tobacco use can greatly facilitate international monitoring and cross-country comparisons.

## Supplementary Material

A Contributorship Form detailing each author’s specific involvement with this content, as well as any supplementary data, are available online at https://academic.oup.com/ntr.

Note: References 21–43 are available as Supplementary Material.

ntab084_suppl_Supplementary_Material

ntab084_suppl_Supplementary_Taxonomy_Form

## References

[1] Ng M, Freeman MK, Fleming TD, et al. Smoking prevalence and cigarette consumption in 187 countries, 1980-2012. JAMA. 2014;311(2):183–192. 24399557 10.1001/jama.2013.284692

[2] Lee YO, Hebert CJ, Nonnemaker JM, Kim AE. Multiple tobacco product use among adults in the United States: cigarettes, cigars, electronic cigarettes, hookah, smokeless tobacco, and snus. Prev Med. 2014;62:14–19. 24440684 10.1016/j.ypmed.2014.01.014

[3] Kasza KA, Ambrose BK, Conway KP, et al. Tobacco-product use by adults and youths in the United States in 2013 and 2014. N Engl J Med. 2017;376(4):342–353. 28121512 10.1056/NEJMsa1607538 PMC5317035

[4] Goodwin RD, Wall MM, Gbedemah M, et al. Trends in cigarette consumption and time to first cigarette on awakening from 2002 to 2015 in the USA: new insights into the ongoing tobacco epidemic. Tob Control. 2018;27(4):379–384. 28801362 10.1136/tobaccocontrol-2016-053601

[5] Sinha DN, Suliankatchi RA, Amarchand R, Krishnan A. Prevalence and sociodemographic determinants of any tobacco use and dual use in six countries of the WHO South-East Asia Region: findings from the demographic and health surveys. Nicotine Tob Res. 2016;18(5):750–756. 26729735 10.1093/ntr/ntv286

[6] National Center for Chronic Disease Prevention and Health Promotion (US) Office on Smoking and Health. Reports of the Surgeon General. *The Health Consequences of Smoking—50 Years of Progress: A Report of the Surgeon General*. Atlanta (GA): Centers for Disease Control and Prevention (US); 2014. 24455788

[7] Soule EK, Pomeranz JL, Moorhouse MD, Barnett TE. Multiple tobacco use and increased nicotine dependence among people with disabilities. Disabil Health J. 2015;8(2):258–263. 25445017 10.1016/j.dhjo.2014.09.004

[8] Bombard JM, Pederson LL, Nelson DE, Malarcher AM. Are smokers only using cigarettes? Exploring current polytobacco use among an adult population. Addict Behav. 2007;32(10):2411–2419. 17490825 10.1016/j.addbeh.2007.04.001

[9] Siddiqi K, Vidyasagaran AL, Readshaw A, Croucher R. A policy perspective on the global use of smokeless tobacco. Curr Addict Rep. 2017;4(4):503–510. 29201593 10.1007/s40429-017-0166-7 PMC5686233

[10] Leavens ELS, Meier E, Brett EI, et al. Polytobacco use and risk perceptions among young adults: the potential role of habituation to risk. Addict Behav. 2019;90:278–284. 30472536 10.1016/j.addbeh.2018.11.003 PMC8049086

[11] Clendennen SL, Loukas A, Creamer MR, Pasch KE, Perry CL. Longitudinal patterns of multiple tobacco and nicotine product use among texas college students: a latent transition analysis. Prev Sci. 2019;20(7):1031–1042. 31302841 10.1007/s11121-019-01031-3 PMC10286813

[12] Simonavicius E, McNeill A, Brose LS. Transitions in smoking and nicotine use from 2016 to 2017 among a UK cohort of adult smokers and ex-smokers. Drug Alcohol Rev. 2020;39:994–1005. 32458503 10.1111/dar.13063

[13] Sung HY, Wang Y, Yao T, Lightwood J, Max W. Polytobacco use of cigarettes, cigars, chewing tobacco, and snuff among US adults. Nicotine Tob Res. 2016;18(5):817–826. 26136525 10.1093/ntr/ntv147 PMC5896811

[14] Shamseer L, Moher D, Clarke M, et al.; PRISMA-P Group. Preferred reporting items for systematic review and meta-analysis protocols (PRISMA-P) 2015: Elaboration and explanation. BMJ. 2015;350:g7647. 25555855 10.1136/bmj.g7647

[15] Centre for reviews and dissemination. Systematic Reviews: CRD’s Guidance for Undertaking Reviews in Health Care. York: University of York; 2006.

[16] World Bank. New country classifications by income level: 2019–2020. https://blogs.worldbank.org/opendata/new-country-classifications-income-level-2019–2020. Accessed June 2020.

[17] Agaku IT, Filippidis FT, Vardavas CI, et al. Poly-tobacco use among adults in 44 countries during 2008-2012: Evidence for an integrative and comprehensive approach in tobacco control. Drug Alcohol Depend. 2014;139:60–70. 24685560 10.1016/j.drugalcdep.2014.03.003

[18] Jawad M, Lee JT, Millett C. The relationship between waterpipe and cigarette smoking in low and middle income countries: Cross-sectional analysis of the global adult tobacco survey. PLoS One. 2014;9(3):e93097. 24664109 10.1371/journal.pone.0093097 PMC3963998

[19] Palipudi KM, Gupta PC, Sinha DN, Andes LJ, Asma S, McAfee T; GATS Collaborative Group. Social determinants of health and tobacco use in thirteen low and middle income countries: evidence from Global Adult Tobacco Survey. PLoS One. 2012;7(3):e33466. 22438937 10.1371/journal.pone.0033466 PMC3306395

[20] Singh A, Ladusingh L. Prevalence and determinants of tobacco use in India: evidence from recent Global Adult Tobacco Survey data. PLoS One. 2014;9(12):e114073. 25474196 10.1371/journal.pone.0114073 PMC4256395

