# Supplementary material for: Global Patterns and Prevalence of Dual and Poly-Tobacco Use: A Systematic Review
Source: Nicotine Tob Res. 2021 Apr 30;23(11):1816–20. doi: 10.1093/ntr/ntab084 (PMC8825763; doi:10.1093/ntr/ntab084)
Supplement: ntab084_suppl_Supplementary_Material [file ntab084_suppl_supplementary_material.docx]

**Supplementary Material**

**Supplement to:**

**Global Patterns and Prevalence of Dual and Poly-Tobacco Use: A Systematic Review**

Daniel Tzu-Hsuan Chen, Charis Girvalaki, Enkeleint A. Mechili PhD, Christopher Millett PhD, Filippos T Filippidis PhD

**Supplementary Table 1.** Example of search strategy of the systematic review

|  |  |
| --- | --- |
| #1 | ((multi* or alternat* or other or poly* or "poly tobacco" or concurrent or dual) adj1 (tobacco or smoking or nicotine or cigarette* or combustible or smoke*)).tw |
| #2 | ("use" or "usage" or product* or consumption or uptake or dependence).tw. |
| #3 | (prevalence or pattern* or type* or trend* or transition* or shift or trajector*).tw. |
| #4 | ("Poly Tobacco" or polytobacco).mp. |
| #5 | #1 and #2 and #3 |
| #6 | #4 or #5 |
| #7  FINAL | remove duplicates from #6 |

Ovid for MEDLINE & Embase & Global Health (adj1: Terms right next to each other in any order)

**Supplementary Table 2.** Summary of the included studies by country and region

| **Region, Country** | **Authors, Year of Publication** | **Survey Year/Period** | **Survey**  **Name** | **Sample**  **Size** | **Dual/Poly**  **tobacco Use** | |
| --- | --- | --- | --- | --- | --- | --- |
|  |  |  |  |  | Dual use Poly use | |
| **South East Asia Region** | |  |  |  |  |  |
| Bangladesh | Sinha D.N. et al., 2016^5^ | 2007 | DHS | 10,400 | 🗸 |  |
|  | Palipudi K.M. et al., 2012^19^ | 2009 | GATS | 9,629 | 🗸 |  |
|  | Agaku I.T. et al., 2014^17^ |  |  |  |  | 🗸 |
|  | Zaman M.M. et al., 2014^21^ | 2010 | N/A | 4,312(men) | 🗸 |  |
| Indonesia | Agaku I.T. et al., 2014^17^ | 2011 | GATS | 8,305 |  | 🗸 |
|  | Sinha D.N. et al., 2016^5^ | 2012 | DHS | 43,852 | 🗸 |  |
| India | Sinha D.N. et al., 2016^5^ | 2005/6 | DHS | 109,041 | 🗸 |  |
|  | Singh A. et al., 2014^20^ | 2009/10 | GATS | 69,296 | 🗸 |  |
|  | Palipudi K.M. et al., 2014^19^ |  |  |  | 🗸 |  |
|  | Agaku I.T. et al., 2014^17^ |  |  |  |  | 🗸 |
|  | Jawad M. et al.,2 014^18^ |  |  |  | 🗸 |  |
| Maldives | Sinha D.N. et al., 2016^5^ | 2009 | DHS | 6,443 | 🗸 |  |
| Nepal | Sinha D.N. et al., 2016^5^ | 2011 | DHS | 10,826 | 🗸 |  |
| Timor-Leste | Sinha D.N. et al., 2016^5^ | 2009/10 | DHS | 11,463 | 🗸 |  |
| Thailand | Palipudi K.M. et al., 2012^19^ | 2009 | GATS | 20,566 | 🗸 |  |
|  | Agaku I.T. et al., 2014^17^ |  |  |  |  | 🗸 |
| **Western Pacific Region** | |  |  |  |  |  |
| China | Palipudi, K. M. 2012^19^ | 2010 | GATS | 13,354 | 🗸 |  |
|  | Agaku, I. T. 2014^17^ |  |  |  |  | 🗸 |
| Malaysia | Agaku I.T. et al., 2014^17^ | 2011 | GATS | 4,250 |  | 🗸 |
| Philippines | Palipudi K.M. et al., 2012^19^ | 2009 | GATS | 9,701 | 🗸 |  |
|  | Agaku, I. T. 2014^17^ |  |  |  |  | 🗸 |
| Viet Nam | Palipudi K.M. et al., 2012^19^ | 2010 | GATS | 9,925 | 🗸 |  |
|  | Jawad M. et al., 2014^18^ |  |  |  | 🗸 |  |
|  | Agaku I.T. et al., 2014^17^ |  |  |  |  | 🗸 |
| **Eastern Mediterranean Region** | |  |  |  |  |  |
| Egypt | Palipudi K.M. et al., 2012^19^ | 2009 | GATS | 20,924 | 🗸 |  |
|  | Jawad M. et al., 2014^18^ |  |  |  | 🗸 |  |
|  | Agaku I.T. et al., 2014^17^ |  |  |  |  | 🗸 |
| **African Region** |  |  |  |  |  |  |
| Nigeria | Agaku I.T. et al., 2014^17^ | 2012 | GATS | 9,765 |  | 🗸 |
| **European** **Region** | |  |  |  |  |  |
| Poland | Palipudi K.M. et al., 2012^19^ | 2009 | GATS | 7,840 | 🗸 |  |
|  | Agaku I.T. et al., 2014^17^ |  |  |  |  | 🗸 |
| France | Agaku I.T. et al., 2014^17^ | 2012 | Eurobarometer | 1,059 |  | 🗸 |
| Belgium | Agaku I.T. et al., 2014^17^ | 2012 | Eurobarometer | 1,051 |  | 🗸 |
| Netherlands | Agaku I.T. et al., 2014^17^ | 2012 | Eurobarometer | 1,014 |  | 🗸 |
| Germany | Agaku I.T. et al., 2014^17^ | 2012 | Eurobarometer | 1,552 |  | 🗸 |
| Italy | Agaku I.T. et al., 2014^17^ | 2012 | Eurobarometer | 1,036 |  | 🗸 |
| Luxembourg | Agaku I.T. et al., 2014^17^ | 2012 | Eurobarometer | 501 |  | 🗸 |
| Denmark | Agaku I.T. et al., 2014^17^ | 2012 | Eurobarometer | 1,019 |  | 🗸 |
| Ireland | Agaku I.T. et al., 2014^17^ | 2012 | Eurobarometer | 1,008 |  | 🗸 |
| United Kingdom | Agaku I.T. et al., 2014^17^ | 2012 | Eurobarometer | 1,331 |  | 🗸 |
| Greece | Agaku I.T. et al., 2014^17^ | 2012 | Eurobarometer | 999 |  | 🗸 |
| Spain | Agaku I.T. et al., 2014^17^ | 2012 | Eurobarometer | 1,004 |  | 🗸 |
| Portugal | Agaku I.T. et al., 2014^17^ | 2012 | Eurobarometer | 1,009 |  | 🗸 |
| Finland | Agaku I.T. et al., 2014^17^ | 2012 | Eurobarometer | 1,003 |  | 🗸 |
| Sweden | Agaku I.T. et al., 2014^17^ | 2012 | Eurobarometer | 1,016 |  | 🗸 |
| Austria | Agaku I.T. et al., 2014^17^ | 2012 | Eurobarometer | 1,031 |  | 🗸 |
| Cyprus | Agaku I.T. et al., 2014^17^ | 2012 | Eurobarometer | 506 |  | 🗸 |
| Czech Republic | Agaku I.T. et al., 2014^17^ | 2012 | Eurobarometer | 1,003 |  | 🗸 |
| Estonia | Agaku I.T. et al., 2014^17^ | 2012 | Eurobarometer | 1,000 |  | 🗸 |
| Hungary | Agaku I.T. et al., 2014^17^ | 2012 | Eurobarometer | 1,021 |  | 🗸 |
| Latvia | Agaku I.T. et al., 2014^17^ | 2012 | Eurobarometer | 1,024 |  | 🗸 |
| Lithuania | Agaku I.T. et al., 2014^17^ | 2012 | Eurobarometer | 1,021 |  | 🗸 |
| Malta | Agaku I.T. et al., 2014^17^ | 2012 | Eurobarometer | 500 |  | 🗸 |
| Slovakia | Agaku I.T. et al., 2014^17^ | 2012 | Eurobarometer | 1,000 |  | 🗸 |
| Slovenia | Agaku I.T. et al., 2014^17^ | 2012 | Eurobarometer | 1,017 |  | 🗸 |
| Bulgaria | Agaku I.T. et al., 2014^17^ | 2011 | Eurobarometer | 1,006 |  | 🗸 |
| Ukraine | Palipudi K.M. et al., 2012^19^ | 2010 | GATS | 8,158 | 🗸 |  |
|  | Agaku I.T. et al., 2014^17^ |  |  |  |  | 🗸 |
| Romania | Agaku I.T. et al., 2014^17^ | 2011 | Eurobarometer | 4,517 |  | 🗸 |
| Russian Federation | Palipudi K.M. et al., 2012^19^ | 2009 | GATS | 11,406 | 🗸 |  |
|  | Jawad M. et al., 2014^18^ |  |  |  | 🗸 |  |
|  | Agaku I.T. et al., 2014^17^ |  |  |  |  | 🗸 |
| Turkey | Palipudi K.M. et al., 2012^19^ | 2008 | GATS | 9,030 | 🗸 |  |
|  | Agaku I.T. et al., 2014^17^ |  |  |  |  | 🗸 |
| **American Region** | |  |  |  |  |  |
| Mexico | Palipudi K.M. et al., 2012^19^ | 2009 | GATS | 13,617 | 🗸 |  |
|  | Agaku I.T. et al., 2014^17^ |  |  |  |  | 🗸 |
| Uruguay | Palipudi K.M. et al., 2012^19^ | 2009 | GATS | 5,581 | 🗸 |  |
|  | Agaku I.T. et al., 2014^17^ |  |  |  |  | 🗸 |
| Brazil | Agaku I.T. et al., 2014^17^ | 2008 | GATS | 39,425 |  | 🗸 |
| Argentina | Agaku I.T. et al., 2014^17^ | 2010 | GATS | 6,645 |  | 🗸 |
| United States | Mumford E.A. et al., 2006^26^ | 1992/93,1995/96,1998/99,2000,2001/02 | TUS-CPS | 374,335 | 🗸 |  |
|  | Backinger C.L. et al., 2008^22^ | 1995/96, 1998, 2000, 2001/02 | TUS-CPS | 552,804 | 🗸 |  |
|  | Tomar S.L. et al., 2010^30^ | 2006 | TUS-CPS | 67,293(men) | 🗸 |  |
|  | Mushtaq N. et al., 2012^27^ | 2010 | BRFSS | 170,114 | 🗸 |  |
|  | Grinberg A. et al., 2016^24^ | 2010/11 | TUS-CPS | 85,545 | 🗸 |  |
|  | Lee Y.O. et al., 2014^2^ | 2012 | NATS | 3,627 | 🗸 | 🗸 |
|  | Sung H.Y. et al., 2016^13^ | 1998, 2000, 2005, 2010 | NHIS | 117,816 | 🗸 | 🗸 |
|  | Wang Y. et al., 2017^31^ | 1998, 2000, 2005, 2010 | NHIS | 123,399 | 🗸 |  |
|  | Roberts M.E. et al., 2017^28^ | 2013/14 | PATH | 32,320 | 🗸 | 🗸 |
|  | Kasza K.A. et al., 2017^3^ | 2013/14 | PATH | 32,320 |  | 🗸* |
|  | Weaver S.R. et al., 2016^32^ | 2014 | TCORS | 5,717 | 🗸 |  |
|  | Syamlal G. et al., 2017^29^ | 2014-2016 | NHIS | 59,690**^※^** |  | 🗸* |
|  | Jones D.M. et al., 2018^25^ | 2015/16 | TCORS | 12,065 | 🗸 |  |
|  | Creamer M.R. et al., 2019^23^ | 2018 | NHIS | 25,417 |  | 🗸* |
| **Total** |  |  |  |  |  |  |
| **48 Countries** | **20 Studies** | **1996-2018** | **11 Datasets** | **2,165,464** |  |  |

* referred as multiple tobacco product use in corresponding studies. ※ sample restricted to working adults.

Abbreviations: DHS: Demographic and Health Surveys; GATS: The Global Adult Tobacco Survey; NSDUH: National Surveys on Drug Use and Health; NATS: National Adult Tobacco Survey; TCORS: Tobacco Products and Risk Perceptions Survey; NHIS: National Health Interview Survey; TUS-CPS: Tobacco Use Supplement-Current Population Survey; PATH: Population Assessment of Tobacco and Health; BRFSS: Behavioral Risk Factor Surveillance System; N/A: locally conducted survey w/o survey name.

**Supplementary Table 3.** Summary of definitions of dual and poly-tobacco use

| **Definitions** |  | **Authors, Year of Publication** |
| --- | --- | --- |
| **Dual /Concurrent Tobacco Use** |  |  |
| Smoking + Smokeless Products^a^ |  | Zaman M.M. et al., 2014; Singh A. et al., 2014;  Palipudi K.M. et al., 2014 |
|  |  |  |
| Cig + One Other Product^b^ |  | Lee Y.O. et al., 2014; Weaver S.R. et al., 2016; Backinger C.L. et al., 2008 |
|  |  |  |
| Cig + SLT^c^ |  | Mumford E.A. et al., 2006; Jones D.M. et al., 2018 Mushtaq N. et al., 2012 |
|  |  |  |
| Any Two Products |  | Sung H.Y. et al., 2016 ;Roberts M.E. et al., 2017 |
|  |  |  |
| Cig + Hookah/Waterpipe |  | Grinberg A. et al., 2016; Jawad M. et al., 2014 |
|  |  |  |
| Cig + Snuff |  | Tomar S.L. et al., 2010 |
|  |  |  |
| Cig + Cigar/Snuff/Chew |  | Wang Y. et al., 2017 |
|  |  |  |
| ≧1 Smoking Product + ≧1 SLT^d^ |  | Sinha D.N. et al., 2016 |
| **Poly /Multiple Tobacco Use** |  |  |
| Any≧2 Products^e^ |  | Sung H.Y. et al., 2016^#^; Agaku I.T. et al., 2014^#^  Syamlal G. et al., 2017*; Kasza, K.A. et al., 2017*  Creamer M.R. et al., 2019* |
|  |  |  |
| Any≧3 Products^f^ |  | Lee Y.O. et al., 2014; Roberts M.E. et al., 2017* |

a Use of one smoked and one smokeless tobacco product

b Current use of cigarette and one other tobacco product

c Current use of cigarettes and smokeless tobacco

d Use of at least one tobacco smoking product and at least one smokeless tobacco product concurrently

e Consuming two or more tobacco products

f Consuming three or more other products

# Poly tobacco use including dual use

* Also referred as multiple tobacco product use

Abbreviations: cig: cigarette, SLT: smokeless tobacco

**Supplementary Table 4.** Prevalence estimates of current single product use among adults by country and region

| **Region, Country** | **Survey Year** | **Single Product Use (%)** | | | | | |
| --- | --- | --- | --- | --- | --- | --- | --- |
|  |  | **Smoking Tobacco** | **Cigarette** | **Cigar/**  **Cigarillo** | **Pipe** | **Hookah/**  **Waterpipe** | **Smokeless Tobacco** |
| **South East Asia** |  |  |  |  |  |  |  |
| Bangladesh^17,19^ | 2009 | 22.9 | 14.5 | 0.4 | 0.4 | 0.7 | 27.1 |
| Indonesia^5^ | 2012 | M: 75.9; F: 2.3 | ― | ― | ― | ― | M: 0.3; F:0.3 |
| India^✝17-20^ | 2009/10 | 14.0 | 6.4 | 0.6 | ― | 0.9 | 29.5 |
| Maldives^5^ | 2009 | M:46.8; F: 4.3 | ― | ― | ― | ― | M:2.9; F:4.0 |
| Nepal^5^ | 2011 | M:17.5; F: 10.2 | ― | ― | ― | ― | M:20.1; F: 4.0 |
| Timor-Leste^5^ | 2009/10 | M:70.5; F:3.2 | ― | ― | ― | ― | M:0.02; F:1.5 |
| Thailand^17^ |  | 23.7 | 29.1 | 0 | 0.1 | 0 | 3.9 |
| **Western Pacific** |  |  |  |  |  |  |  |
| China^17,19^ | 2010 | 28.1 | 29.0 | 0.1 | 0.5 | 0.4 | 0.5 |
| Malaysia^17^ | 2011 | ― | 24.1 | 0.2 | 0.2 | 0.6 | 0.7 |
| Philippines^17,19^ | 2009 | 28.9 | 28.3 | 0.3 | 0.1 | 0 | 2.0 |
| Viet Nam^17-19^ | 2010 | 23.7 | 20.6 | 0 | 0.1 | 6.4 | 1.3 |
| **Eastern Mediterranean** | |  |  |  |  |  |  |
| Egypt^17-19^ | 2009 | 19.4 | 16.4 | 0 | 0 | 3.3 | 2.6 |
| **African** | |  |  |  |  |  |  |
| Nigeria^17^ | 2012 | ― | 4.7 | 0.5 | 0.3 | 0.3 | 1.90 |
| **European** | |  |  |  |  |  |  |
| Poland^17,19^ | 2009 | 30.3 | 31.7 | 0.5 | 0.2 | ― | 0.5 |
| France^17^ | 2012 | ― | 32.4 | 1.4 | 0.4 | 7.1 | 0.8 |
| Belgium^17^ | 2012 | ― | 31.6 | 2.0 | 0.6 | 4.7 | 0.9 |
| Netherlands^17^ | 2012 | ― | 28.9 | 2.9 | 1.1 | 5.1 | 0.4 |
| Germany^17^ | 2012 | ― | 30.1 | 0.9 | 0.7 | 5.0 | 1.8 |
| Italy^17^ | 2012 | ― | 26.9 | 1.1 | 0.6 | 2.0 | 1.7 |
| Luxembourg^17^ | 2012 | ― | 28.1 | 2.5 | 0.5 | 7.8 | 1.4 |
| Denmark^17^ | 2012 | ― | 27.4 | 0.7 | 3.0 | 8.4 | 3.2 |
| Ireland^17^ | 2012 | ― | 33.2 | 0.5 | 0.2 | 0.9 | 1.6 |
| United Kingdom^17^ | 2012 | ― | 33.8 | 0.8 | 0.6 | 3.6 | 1.1 |
| Greece^17^ | 2012 | ― | 46.6 | 0.3 | 0.2 | 2.9 | 0.1 |
| Spain^17^ | 2012 | ― | 38.3 | 0.7 | 0.4 | 2.9 | 0.3 |
| Portugal^17^ | 2012 | ― | 26.6 | 0 | 0.2 | 1.9 | 2.7 |
| Finland^17^ | 2012 | ― | 29.6 | 2.3 | 0.7 | 1.5 | 3.4 |
| Sweden^17^ | 2012 | ― | 12.4 | 0.5 | 0.9 | 2.8 | 17.7 |
| Austria^17^ | 2012 | ― | 35.4 | 0.8 | 0.2 | 8.5 | 4.3 |
| Cyprus^17^ | 2012 | ― | 35.4 | 0.8 | 0.2 | 8.5 | 1.2 |
| Czech Republic^17^ | 2012 | ― | 31.9 | 0.5 | 0.4 | 7.9 | 1.4 |
| Estonia^17^ | 2012 | ― | 28.3 | 1.1 | 1.0 | 7.6 | 3.6 |
| Hungary^17^ | 2012 | ― | 36.8 | 0.4 | 0.3 | 2.4 | 0.4 |
| Latvia^17^ | 2012 | ― | 39.9 | 1.3 | 1.0 | 11.5 | 0.7 |
| Lithuania^17^ | 2012 | ― | 32.0 | 0.5 | 0.6 | 9.0 | 0.6 |
| Malta^17^ | 2012 | ― | 30.4 | 1.0 | 0.9 | 2.8 | 3.5 |
| Slovakia^17^ | 2012 | ― | 23.3 | 0.6 | 0.2 | 4.2 | 2.2 |
| Slovenia^17^ | 2012 | ― | 29.9 | 0 | 0.4 | 2.0 | 1.1 |
| Bulgaria^17^ | 2011 | ― | 38.9 | 0 | 0 | 1.4 | 0.1 |
| Ukraine^17,19^ | 2010 | 28.9 | 29.6 | 0.7 | 0.2 | 2.1 | 0.3 |
| Romania^17^ | 2011 | ― | 27.2 | 0.7 | 0.2 | 0.4 | 0.3 |
| Russian Federation^17-19^ | 2009 | 39.2 | 39.2 | 1.3 | 0.8 | 3.8 | 0.6 |
| Turkey^17,19^ | 2008 | 31.2 | 32.7 | 0.7 | 0.3 | 2.3 | 0 |
| **American** | |  |  |  |  |  |  |
| Mexico^17,19^ | 2009 | 15.9 | 15.9 | 0.3 | 0.1 | 0 | 0.3 |
| Uruguay^17,19^ | 2009 | 24.9 | 29.4 | 0.1 | 0.2 | 0.1 | 0 |
| Brazil^17^ | 2008 | ― | 19.4 | 0.1 | 0.3 | 0.1 | 0.4 |
| Argentina^17^ | 2010 | ― | 22.7 | 0.3 | 0.1 | 0 | 0.2 |
| United States^23^ | 2018 | 16.5 | 13.7 | 3.9 | 1.0 | 1.0 | 2.4 |
|  |  |  |  |  |  |  |  |

Note:

1.Table summarises prevalence estimates weighted from various studies and surveyed years, and therefore, figures may not be directly comparable between each usage groups.

2.Table presents most recent prevalence estimates available of each country and product use.

✝ % for bidi use in India= 9.2%; ― data not available.

Abbreviations: M: male; F: female.

**Supplementary Table 5.** Prevalence estimates of current tobacco product use among adults in the US

| **Authors, Year of Publication** | **Survey Year** | **Single Product Use (%)** | | | |  | **Dual Tobacco Use (%)** | | |  | **Poly Tobacco Use (%)** | |
| --- | --- | --- | --- | --- | --- | --- | --- | --- | --- | --- | --- | --- |
|  |  | Cigarette | Cigar | Hookah/ Waterpipe | Smokeless Tobacco |  | Cig+SLT | Cig+hookah/waterpipe | Cig+cigars |  | Any≧2 products^††^ | Any≧3 products^‡‡^ |
| Mumford E.A. et al., 2006 | 2001/02 | 21.9 | ― | ― | 1.6 |  | 0.32 | ― | ― |  | ― | ― |
| Backinger C.L. et al., 2008^a^ | 2001/02 | 20.55 | ― | ― | ― |  | 2.15* | ― | 3.49 |  | 1.19 | ― |
| Tomar S.L. et al., 2010^b^ | 2006 | 19.8 | ― | ― | 2.1† |  | 6.6† | ― | ― |  | ― | ― |
| Mushtaq N. et al., 2012 | 2010 | M:19.07; F:15.14 | ― | ― | M:5.78; F:0.82 |  | M:1.62; F:0.35 | ― | ― |  | ― | ― |
| Grinberg A. et al., 2016 | 2010/11 | 16.7 | ― | M:0.8; F:0.4 | ― |  | ― | 3.4 | ― |  | ― | ― |
| Lee Y.O. et al., 2014^c^ | 2012 | 24.2 | 8.2 | 5.5 | Snus:4;Chew: 5.7 |  | 2.8 | 4.2 | 5 |  | ― | 2.4 |
| Sung H.Y. et al., 2016 | 2010 | 19.3 | 2.5 | ― | Snuff:2;Chew: 1.1 |  | 2.2†; 1.5‡ | ― | 5.2 |  | 7.6 | ― |
| Wang Y. et al., 2017 | 2010 | 24.2 | 5 | ― | ― |  | 2.1†; 1.5‡ | ― | 5 |  | ― | ― |
| Roberts M.E. et al., 2017^d^ | 2013/14 | 22.5 | 3.6 | 2.2 | 3.3 |  | ― | ― | ― |  | 10.3 | ― |
| Kasza K.A. et al., 2017^e^ | 2013/14 | 22.5 | 3.6 | 2.2 | 3.3 |  | 4 | 6 | 4 |  | 37.8¶ | 15.3¶ |
| Weaver S.R. et al., 2016^f^ | 2014 | 16.6 | 1.8 | 1.2 | 2.1 |  | 3.5 | 2 | 2.7 |  | ― | ― |
| Syamlal G. et al., 2017^g^ | 2014-2016 | 15.4 | ― | ― | 3 |  | ― | ― | ― |  | 4.6 | ― |
| Jones D.M. et al., 2018 | 2015/16 | 13.6 | ― | ― | ― |  | 0.8 | ― | ― |  | ― | ― |
| Creamer M.R. et al., 2019 | 2018 | 13.7 | 3.9^§^ | 1 | 2.4 |  | ― | ― | ― |  | 3.7 | ― |
|  |  |  |  |  |  |  |  |  |  |  |  |  |

Note:

1.Current use was determined as participants who had smoked or used the product in the previous 30 days.

2.Table summarises prevalence estimates weighted from various studies and surveyed years, and therefore, figures may not be directly comparable between each usage groups.

3.Table presents most recent prevalence estimates available of each country and product use.

Abbreviations: cig: cigarettes; SLT: smokeless tobacco; M: male; F: female.

*snuff and chewing tobacco combined; †snuff; ‡ chewing; ¶ prevalence estimates among tobacco users; § cigars, Cigarillos and filtered cigars combined; ― data not available;

†† consuming two or more tobacco products; ‡‡ consuming three or more other products

a: Cig+pipe: 0.78%; Cig+snuff: 0.97%; Cig+chewing: 1.36%.

b: Prevalence estimates restricted to males.

c: Cig+snus: 2.6%.

d: Cigarillo: 4.4%; pipe: 0.9%.

e: Cigarillo: 4.4%; filtered cigars: 2%; pipe: 1.1%; snus: 0.8%; dissolvable: 0.1%; cig+cigarillos: 5%; cig+filtered cigars: 3%; cigar+cigarillos: 3%; cigar+SLT: 1%; cigar+hookah: 1%; cigarillos+hookah: 1%; cigars+cigarillos+flitered cigar: 1%; cig+cigars+cigarillos: 3%; cig+cigarillos+hookah: 2%; cig+cigarillos+filtered cigar: 1%; cig+cigar+cigarillos+filtered cigar: 1%.

f: Cigarillo: 2.8%; Cig+cigarillos: 9.4%; Cig+snus:1.2%.

g: Prevalence estimates restricted to working adults.

**Supplementary Figure1.** Map of the 48 countries included in the review

**
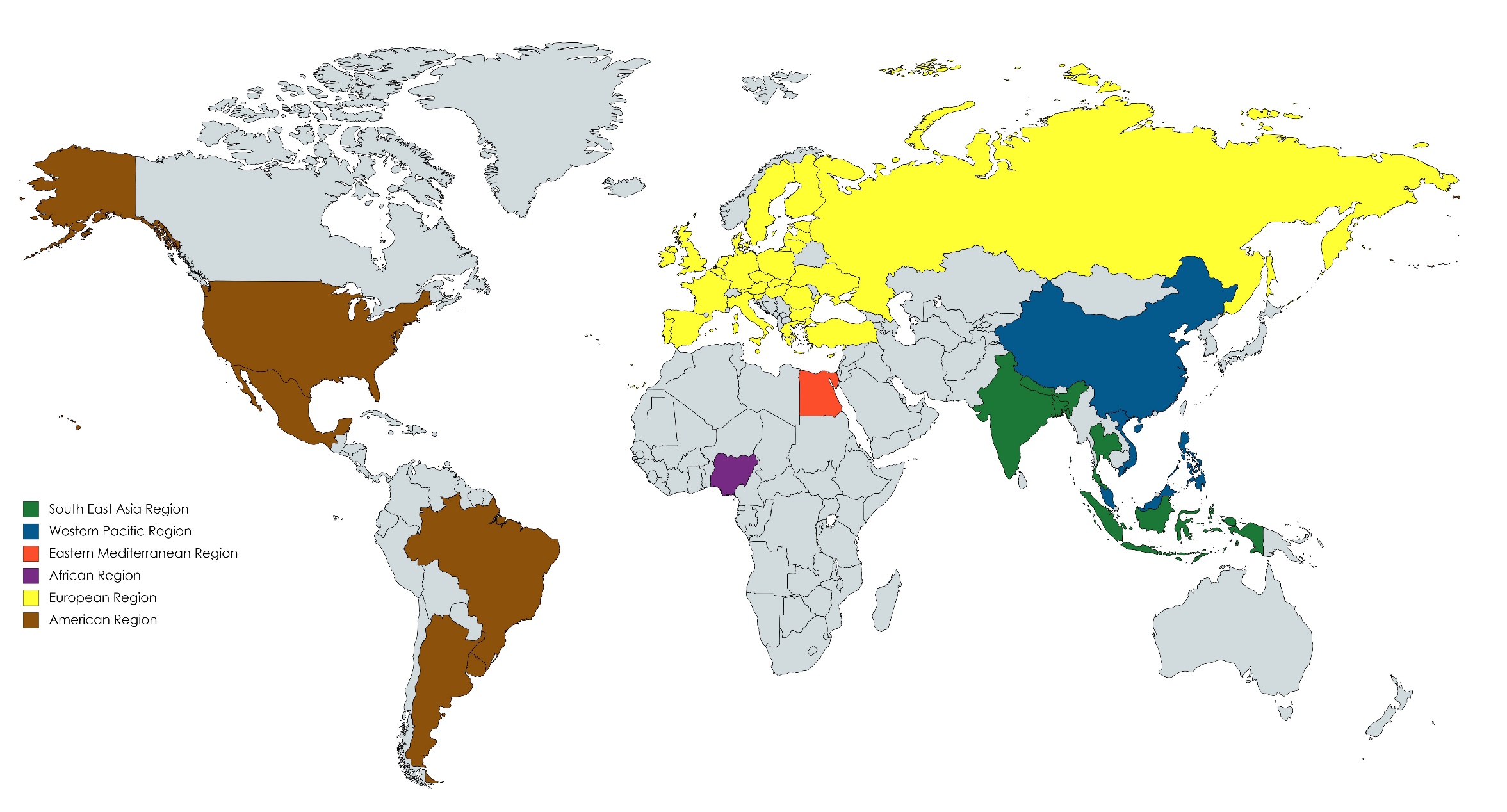
**

**SUPPLEMENTARY REFERENCE**

21. Zaman MM, Bhuiyan MR, Huq SM, Rahman MM, Sinha DN, Fernando T. Dual use of tobacco among Bangladeshi men. *Indian J Cancer.* 2014;51 Suppl 1(Suppl 1):S46-49.

22. Backinger CL, Fagan P, O'Connell ME, et al. Use of other tobacco products among U.S. adult cigarette smokers: prevalence, trends and correlates. *Addict Behav.* 2008;33(3):472-489.

23. Creamer M.R., Wang T.W., Babb S., et al. Tobacco Product Use and Cessation Indicators Among Adults - United States, 2018. *MMWR Morb Mortal Wkly Rep.* 2019;68:1013-1019.

24. Grinberg A, Goodwin RD. Prevalence and correlates of hookah use: a nationally representative sample of US adults ages 18-40 years old. *Am J Drug Alcohol Abuse.* 2016;42(5):567-576.

25. Jones DM, Popova L, Weaver SR, Pechacek TF, Eriksen MP. A National Comparison of Dual Users of Smokeless Tobacco and Cigarettes and Exclusive Cigarette Smokers, 2015-2016. *Nicotine Tob Res.* 2018;20(suppl_1):S62-s70.

26. Mumford EA, Levy DT, Gitchell JG, Blackman KO. Smokeless tobacco use 1992–2002: trends and measurement in the Current Population Survey-Tobacco Use Supplements. *Tobacco Control.* 2006;15(3):166.

27. Mushtaq N, Williams MB, Beebe LA. Concurrent use of cigarettes and smokeless tobacco among US males and females. *J Environ Public Health.* 2012;2012:984561.

28. Roberts ME, Doogan NJ, Stanton CA, et al. Rural Versus Urban Use of Traditional and Emerging Tobacco Products in the United States, 2013-2014. *Am J Public Health.* 2017;107(10):1554-1559.

29. Syamlal G, King BA, Mazurek JM. Tobacco Use Among Working Adults - United States, 2014-2016. *MMWR Morb Mortal Wkly Rep.* 2017;66(42):1130-1135.

30. Tomar SL, Alpert HR, Connolly GN. Patterns of dual use of cigarettes and smokeless tobacco among US males: findings from national surveys. *Tob Control.* 2010;19(2):104-109.

31. Wang Y, Sung HY, Yao T, Lightwood J, Max W. Infrequent and Frequent Nondaily Smokers and Daily Smokers: Their Characteristics and Other Tobacco Use Patterns. *Nicotine Tob Res.* 2018;20(6):741-748.

32. Weaver SR, Majeed BA, Pechacek TF, Nyman AL, Gregory KR, Eriksen MP. Use of electronic nicotine delivery systems and other tobacco products among USA adults, 2014: results from a national survey. *Int J Public Health.* 2016;61(2):177-188.

33. World Meters. Population by Gender, Age, Fertility Rate, Immigration. https://www.worldometers.info/world-population/world-population-gender-age.php. Accessed July 2020.

34. Global Adult Tobacco Survey Collaborative Group. *Tobacco Questions for Surveys: A subset of key questions from the Global Adult Tobacco Survey (GATS).* Second Edition ed. Atlanta GA: Centers for disease Control and Prevention; 2011.

35. Cahn Z, Drope J, Hamill S, et al. *The Tobacco Atlas: Sixth Edition.* Atlanta: American Cancer Society, Inc; 2018.

36. Giovino GA, Mirza SA, Samet JM, et al. Tobacco use in 3 billion individuals from 16 countries: an analysis of nationally representative cross-sectional household surveys. *Lancet.* 2012;380(9842):668-679.

37. Partos TR, Gilmore AB, Hitchman SC, Hiscock R, Branston JR, McNeill A. Availability and Use of Cheap Tobacco in the United Kingdom 2002-2014: Findings From the International Tobacco Control Project. *Nicotine Tob Res.* 2018;20(6):714-724.

38. Filippidis FT, Jawad M, Vardavas CI. Trends and Correlates of Waterpipe use in the European Union: Analysis of Selected Eurobarometer Surveys (2009-2017). *Nicotine Tob Res.* 2019;21(4):469-474.

39. National Cancer Institute (NCI), Centers for Disease Control and Prevention (CDC). Smokeless tobacco and public health: a global perspective. U.S. department of health and human services, centers for disease control and prevention and national institutes of health, national cancer institute.: NIH Publication No. 14-7983; 2014.

40. World Health Organization. *WHO report on the global tobacco epidemic, 2019: offer help to quit tobacco use.* Geneva: World Health Organization; 2019.

41. Sinha DN, Rizwan SA, Aryal KK, Karki KB, Zaman MM, Gupta PC. Trends of Smokeless Tobacco use among Adults (Aged 15-49 Years) in Bangladesh, India and Nepal. *Asian Pac J Cancer Prev.* 2015;16(15):6561-6568.

42. Klesges RC, Ebbert JO, Morgan GD, et al. Impact of differing definitions of dual tobacco use: implications for studying dual use and a call for operational definitions. *Nicotine Tob Res.* 2011;13(7):523-531.

43. Zaatari GS, Bazzi A. Impact of the WHO FCTC on non-cigarette tobacco products. *Tob Control.* 2019;28(Suppl 2):s104-s112.
